# Comparative characterization of bacterial communities in geese fed all-grass or high-grain diets

**DOI:** 10.1371/journal.pone.0185590

**Published:** 2017-10-03

**Authors:** Qi Xu, Xiaoya Yuan, Tiantian Gu, Yang Li, Wangcheng Dai, Xiaokun Shen, Yadong Song, Yang Zhang, Wenming Zhao, Guobin Chang, Guohong Chen

**Affiliations:** 1 Key Laboratory of Animal Genetics and Breeding and Molecular Design of Jiangsu Province, Yangzhou University, Yangzhou, PR, China; 2 Waterfowl Institute of Zhenjiang City, Dantu, China; Universite Paris-Sud, FRANCE

## Abstract

**Background:**

Gut microbial composition is dependent on diet. Geese are herbivores and can digest crude fibre, but the relationship between composition of the microbiota and a fibre-rich diet in geese is not well understood.

**Results:**

Here, caecal and faecal samples were collected simultaneously from all-grass-fed geese and high-grain-fed geese and the hypervariable V3–V4 regions of the bacterial 16S rRNA gene were sequenced. The results was identified that high-grass-fed geese possessed significantly higher alpha diversity both in caecum and faeces compared with that in all-grain-fed geese. In addition, the composition of dominant bacterium occurred remarkable shifting due to different diet patterns, Firmicutes were more abundant in all-grass-fed geese, whereas Bacteroidetes were abundant in high-grain-fed geese. Fusobacteria and Deferribacteres were obviously present in high-grain-fed geese and few in all-grass-fed geese. Most importantly, some specific microorgnisms such as Ruminococcaceae, Lachnospiraceae and Bacteroidaceae which may associated with cellulose-degrading that were characterized to show distinctly diverse between the two diet patterns. PICRUSt analysis revealed the metabolic pathways such as carbohydrate and amino acid metabolism were overrepresented in all-grass-fed geese.

**Conclusions:**

In conclusion, Firmicutes and Bacteroidetes were identified abundantly when the geese was fed with all-grass feed and high-grain feed, respectively. And Ruminococcaceae, Lachnospiraceae and Bacteroidaceae were recognized as main cellulose-degrading bacteria in the geese. The functional profiles of gut microbiota revealed the dominant microbiota communities were involved mainly in the carbohydrate metabolism in all-grass-fed geese.

## Introduction

The gut microbiota is the community of microorganisms that colonize the gut of a host organism. Microorganisms colonize the gut via a very complex process of interactions between the microorganisms and the host. The host provides a place for the survival and evolution of microorganisms, and in return, the microbiota benefits the host by providing essential nutrients, as well as stimulating growth performance [1, 2]. The mammalian gut microbiota has been widely surveyed, and its diversity, structure, and function have been found to be mainly shaped by adaptation to diet [3–5]. Highly diverse cellulolytic obligate anaerobes, such as lineages within Bacteroidales, Fibrobacterales, and Spirochaetales colonize the gastrointestinal tracts of most herbivores, providing microbial fermentation to enhance nutrient absorption [6, 7]. Previous research has shown that, the improvements in cellulose degradation would have a favourable impact on animal productivity in herbivores. *Ruminococcus* and *Fibrobacter* are important members of the rumen microbial community that enable the host to degrade and utilize fibrous plant materials efficiently as nutrients [8–10].

The goose (*Anas cygnoides*) is a commercially important food source that is widely cultivated in China [11]. Under intensive systems, geese are fed diets consisting of large proportions of grains to support rapid weight gain. Although these feeding practices may be helpful in enhancing cost-efficiency in the short term, it is unclear whether such intensive systems affect intestinal microbial structure or gastrointestinal health. After all, geese are basically herbivores under natural conditions.

It is well known that the goose has a strong ability to digest crude fibre [12–14]. However, the goose is genetically deficient in cellulose-digesting enzymes, and digests fibre mainly by microorganism fermentation in the caecum [15–17]. Therefore, it is important to study the diverse bacterial community to reveal the molecular mechanisms underlying this strong ability of the goose to digest crude fibre. Liu [15] analysed the goose caecal bacterial community by denaturing gradient gel electrophoresis (DGGE), and observed an increased diversity in caecal microbiota with an increase in dietary crude fibre levels. They further found that uncultured *Clostridiaceae sp*., *uncultured Treponema sp*., *Cellulomonas sp*., uncultured *Bacteroides sp*., and uncultured *Eubacteriaceae* bacterium were the major cellulose-degrading bacteria [15]. However, the DGGE approach only isolates small DNA fragments, and this might have resulted in the loss of some useful sequence information. Furthermore, this approach usually displays DNA fragments from dominant communities due to its low resolution. Recently, with the development of next-generation sequencing technologies, 16S rRNA gene amplicon deep sequencing has been applied widely to investigate the microbiota [1, 18, 19].

In the present study, we investigated the effects of different diets (all-grass diet and high-grain diet) on the microbiota of geese, using paired-end MiSeq sequencing to characterize the bacterial community of the caecum and faeces. These data may identify bacterial taxa that are associated with the digestion of crude fibre.

## Materials and methods

### Ethics statement

The animal experiment was reviewed and approved by the Institutional Animal Care and Use Committee of Yangzhou University (approval number:151–2014). Procedures were performed in accordance with the Regulations for the Administration of Affairs Concerning Experimental Animals (Yangzhou University, China, 2012) and the Standards for the Administration of Experimental Practices (Jiangsu, China, 2008).

### Animals and sample collection

A total of forty 35-day-old Yangzhou goslings weighing an average of 1404.05 g were selected from the breeding farm of the Yangzhou Goose Co. Ltd (Yangzhou, China). The goslings were randomly assigned to two groups. One group was fed by rotation grazing on a pasture of perennial ryegrass and Chinese trumpet creeper (220 g crude fibre/kg DM), and the other group was fed a high-grain diet consisting mainly of maize and soybean meal (100 g crude fibre/kg DM, produced by Changzhou Chia Tai Co., Ltd). During the experiment, geese were fed *ad libitum* with the grass diet or the high-grain diet. The experimental period lasted 5 weeks, the body weight was shown in S6 Table, the body weight of 70-day-old geese on the high-grain diet was higher than that of geese on the grass diet (*P*<0.05). The female geese from two groups were placed individually in a cage and their defecation observed from a distance. Once the faeces were excreted from the goose, the faece was selected immediately from the central portion of each cage to minimize contamination. After collection, samples were stored on ice until frozen at -80°C.

Following this, the same geese were slaughtered immediately by anesthetizing them with sodium pentobarbital for intestinal sampling. The abdominal cavity was opened by a midline incision, and the intestinal tract was removed carefully. A segment of the caecum was tied at both ends and the contents collected on ice, and then stored at –80°C until DNA extraction.

### DNA extraction and PCR amplification of 16S rRNA gene

Microbial genomic DNA was extracted from each samples using the E.Z.N.A.^®^ DNA Kit (Omega Bio-tek, Norcross, GA, U.S.) according to the manufacturer’s protocols. DNA extraction was carried out using the bead-beating method, with a mini-bead beater. The frozen samples were placed into bead tubes, and then added 1ml Buffer of SLX Mlus to the samples, incubated at 70°C for 10min. The samples were beaten for 90 s at 5000 rpm (maximum speed) in a Mini-Beadbeater^TM^ (Biospec Products Inc., Bartlesville, OK). The solution was precipitated with ethanol, and the pellets were suspended in 50 μL Tris-EDTA buffer. The DNA concentration was determined using a NanoDrop spectrophotometer (NanoDrop, USA).

The V3–V4 regions of the bacterial 16S rRNA gene were amplified using PCR (95R (95 (95h ethanol, and the pellets were suspen 30 s, 55°C for 30 s, 72°C for 45 s, and a final extension at 72°C for 10 min) using the primers [20] 338F 5′- ACTCCTACGGGAGGCAGCA-3′and 806R 5′- GGACTACHVGGGTWTCTAAT-3′. The barcode is an eight-base sequence unique to each sample. PCR reactions were performed in triplicate in a 20-μL mixture containing 4 μL of 5× Fast Pfu buffer, 2 μL of 2.5 mM dNTPs, 0.4 mL of each primer (5μM), 0.4 μL of Fast Pfu polymerase, and 10 ng of template DNA.

### Illumina MiSeq sequencing

The amplicons were examined on a 2% (w/v) agarose gel. The band was extracted and purified with the AxyPrepDNA Gel (Axygen, USA) according to the manufacturer’s instructions, and quantified using QuantiFluor™uantiFluorrding to the manufaamplicons were pooled in equimolar amounts and paired-end sequenced (2uenced (2end sIllumina MiSeq platform (Majorbio, China) according to the standard protocols.

### Processing and bioinformatics analysing of sequencing data

Raw FASTQ files were de-multiplexed and quality-filtered using QIIME (version1.17) with the following criteria [21] (i) The 300-bp reads were truncated at any site that obtained an average quality score of <20 over a 50-bp sliding window, and truncated reads shorter than 50 bp were discarded. (ii) Only overlapping sequences longer than 10 bp were assembled according to their overlapped sequence. Reads that could not be merged were discarded. (iii) Reads with exact barcode matching, no more than two-nucleotide mismatch in primer matching were left, while reads containing ambiguous characters were removed. OTUs at or above the 97% similarity cutoff were clustered using UPARSE software (version 7.1, http://drive5.com/uparse) [22]. Taxonomic classification of OTUs was carried out using the Ribosomal Database Project (RDP) Classifier [23]. Rarefaction analysis based on MOTHUR (version 1.30.1) was conducted to reveal alpha diversity indices, including the Chao, ACE, and Shannon diversity indices [24]. Beta diversity analysis was performed using UniFrac to compare the results from principal component analysis (PCA) [25]. Linear discriminant analysis (LDA) coupled with effect size (LEfSe) was performed to identify bacterial taxa that were differentially represented between groups at the genus level or higher taxonomic levels [26], and only taxa meeting an LDA significance threshold ≥ 2 and the parameter of LEfSe for p valuevalue owere adopted. The functional profiles of microbial communities were predicted using PICRUSt [27]. The R packages ‘heatmap’ and ‘vegan’ were used for redundancy analysis (RDA) and plotting (including generation of the PCA and heat map figures) [28]. To compare bacterial communities of different geese, We downloaded and analysed the data of goose microbiota from NCBI (ERR011376). The sequences satisfied the inclusion criteria of sequence length>500 bp, and data sets containing>50 sequences.

### Statistical analysis

Species richness and alpha diversity were analysed by two-way analysis of variance (ANOVA). Each individual OTU was analysed for effects of diet by ANOVA. The ANOVA analysis was performed using SPSS 17.0 software. *P*<0.05 was considered statistically significant. Differentially abundant OTUs were analysed using Metastats software [29].

## Results

### General DNA sequencing observations

**Fig 1:**
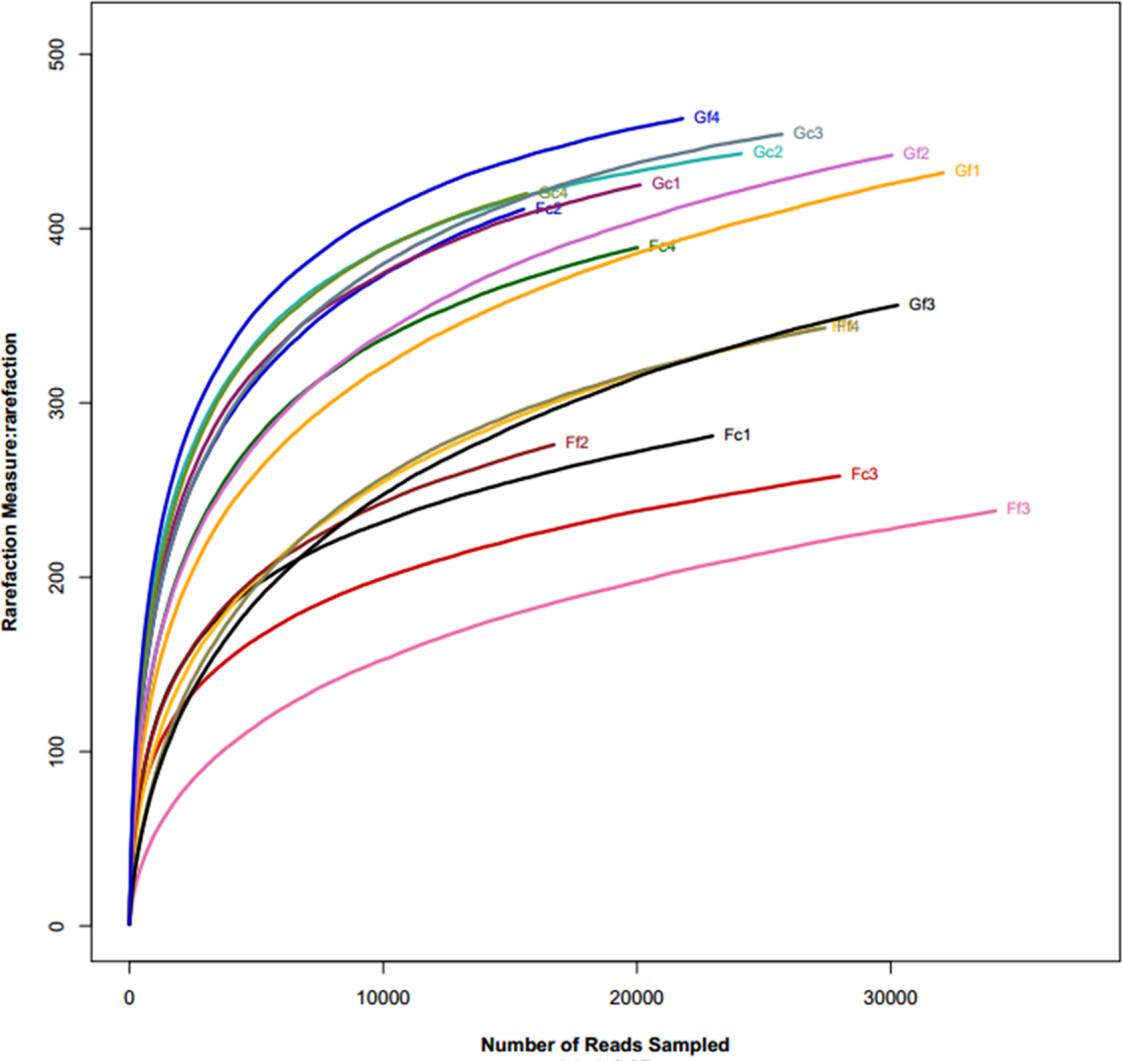
Rarefaction analysis of the different samples. Rarefaction curves of OTUs clustered at 97% sequence identity across different samples. From the all-grass-fed (G) and high-grain-fed (F) groups, both faecal (f) and caecal (c) samples were used from four individuals (1–4).

To characterize the bacterial lineages present in the faecal and caecal microbiota of all-grass-fed geese and high-grain-fed geese, we performed Illumina sequencing of the V3gquehypervariable regions of 16S rRNA gene with the PE300 platform. We generated a dataset consisting of 495,646 filtered, high-quality, classifiable 16S rRNA gene sequences with a mean average (eanith a30,977±5,218 sequences per sample (**S1 and S2 Tables**). The sequence data file was submitted to the Short read archive at NCBI (Accession NO. SRR3711176). The quality-filtered sequences were clustered OTUs at the 97% similarity cut-off. The singletons were removed, and the OTUs including three sequences were retained. A total of 391,736 reads were assigned to 933 non-singleton OTUs at the 97% similarity cut-off, which were assigned into 149 taxa (genus level). The rarefaction curves tended to approach the saturation plateau (**Fig 1**). Good’s coverage estimates indicated that a large part of the diversity in all samples had been captured, with the average coverage being 97.06%(S.D.1.47%).

### Microbial richness and biodiversity

We then compared the microbial α-diversity in faecal and caecal samples between the all-grass-fed and high-grain-fed groups, including microbial richness estimated by the ACE and Chao1 indices, and diversity assessed by the Shannon and Simpson indices (**Table 1**). At the 0.03 dissimilarity level, we found significant differences in richness between different diet-fed geese in both faecal and caecal samples (*P*<0.01 or *P*<0.05), with higher microbial richness in all-grass-fed geese. The caecum exhibited significantly high microbial diversity from the indices of Shannon and Simpson when compared with the faeces (*P*<0.05).

**Table 1:** Effects of diet and sample type on the diversity of the bacterial community at the 3% dissimilarity level.

Diet	Sample type	ACE	Chao1	Shannon	Simpson
All-grass diet	Faeces	497.0±34.4^Aa^	493.8±40.3^Aa^	3.7±1.1^ABac^	0.10±0.10^Aab^
	Caecum	476.3±12.74^ABa^	481.3±13.0^Aa^	4.5±0.1^Aa^	0.03±0.01^Ab^
High-grain diet	Faeces	372.3±45.3^Bb^	373.8±38.6^Bb^	2.5±1.0^Bbc^	0.27±0.22^Aa^
	Caecum	398.8±75.6^ABb^	399.8±74.1^ABb^	3.9±0.4^ABa^	0.05±0.02^Ab^
		*P*			
Diet		<0.01	<0.01	<0.05	0.168
Sample type		0.906	0.778	<0.05	<0.05
Diet * Sample type		0.342	0.427	0.475	0.283

Different capital letters indicate very significant differences between respective means (*P*<0.01), while different lowercase letters indicate significant differences (*P*<0.05).

**Fig 2:**
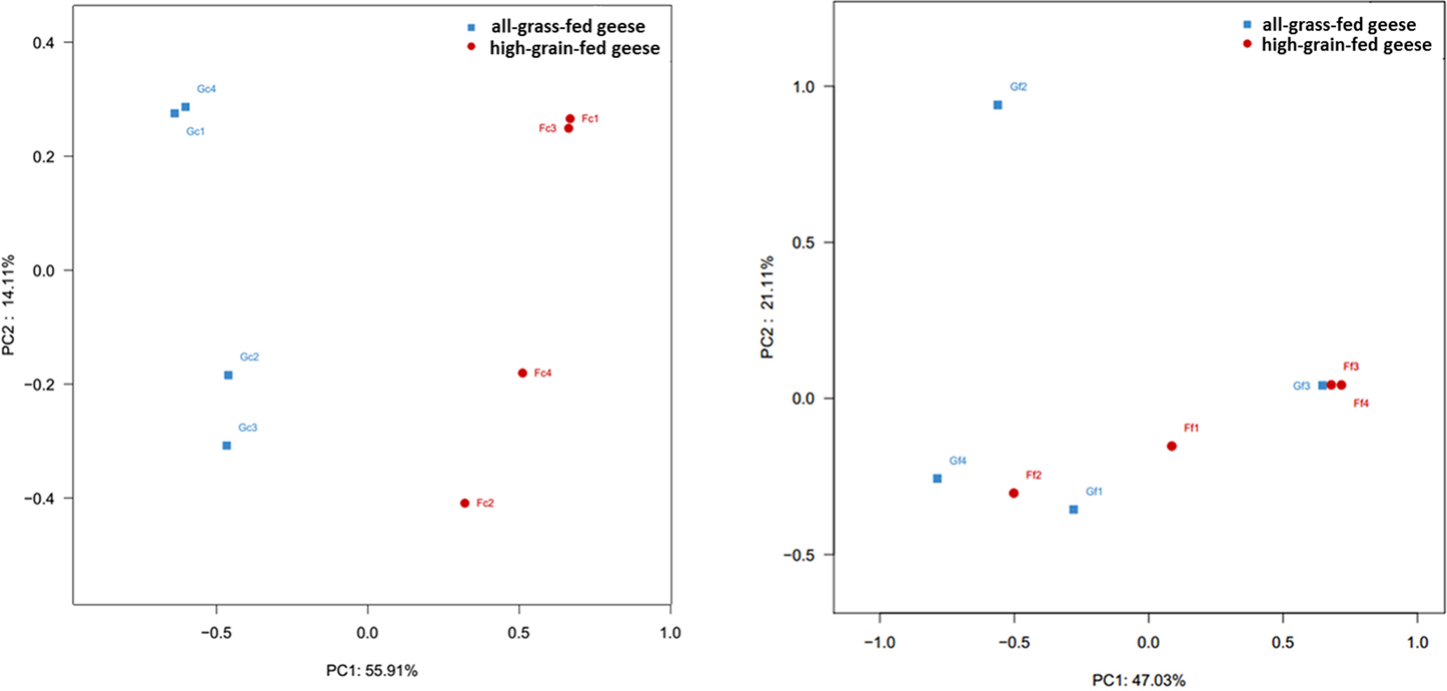
Principal component analysis (PCA) of bacterial communities in caecal or faecal samples of geese. Scatterplot of PCA scores depicting the variance of fingerprints derived from different bacterial communities. Principal components (PCs) 1 and 2 accounted for 55.91% and 14.11% of the variance, respectively. (a) Unweighted PCA scores of caecal microbiota; (b) unweighted PCA scores of faecal microbiota.

To compare community compositions across the different samples, The unweighted UniFrac metric was calculated to assess β-diversity by MOTHUR software. The results indicated that unweighted UniFrac was able to distinctly identify caecum microbiota from geese fed the two different diets (**Fig 2(A)**), and showed little differentiation in faecal samples (**Fig 2(B)**). Principal coordinate analysis revealed that principal component analysis (PCA) axis 1 accounted for 55.91% of the variation and PCA axis 2 for 14.11% of the variation in the caecum.

### Differences in caecal bacterial communities between all-grass-fed geese and high-grain-fed geese

**Fig 3:**
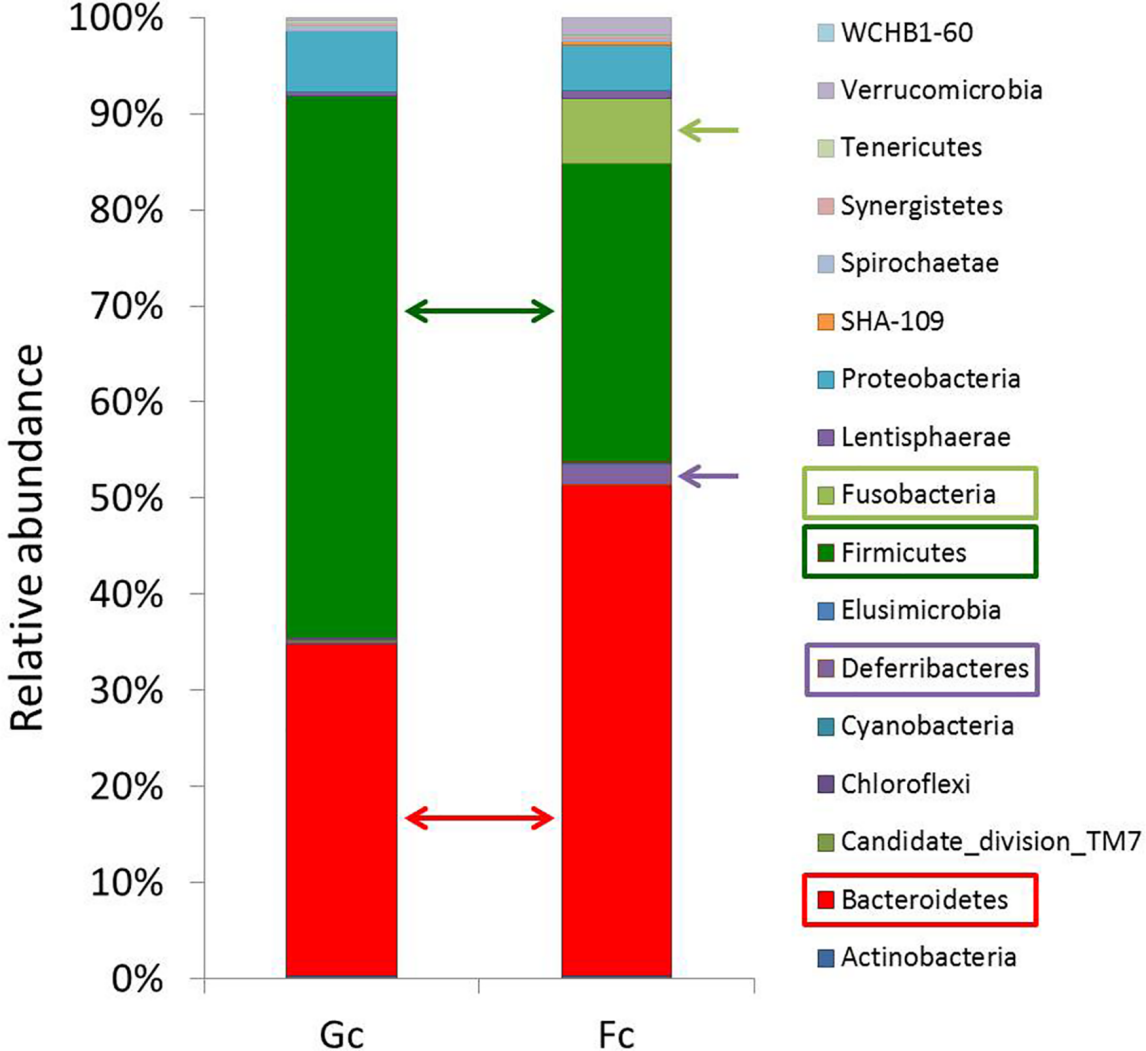
Distribution of the phyla averaged across dietary treatment in caecal samples from geese. Data were analysed by RDP classifier v.2.2. Bacterial communities in caecal samples from all-grass-fed geese (Gc) and high-grain-fed geese (Fc). Arrows indicate different phyla.

**Fig 4:**
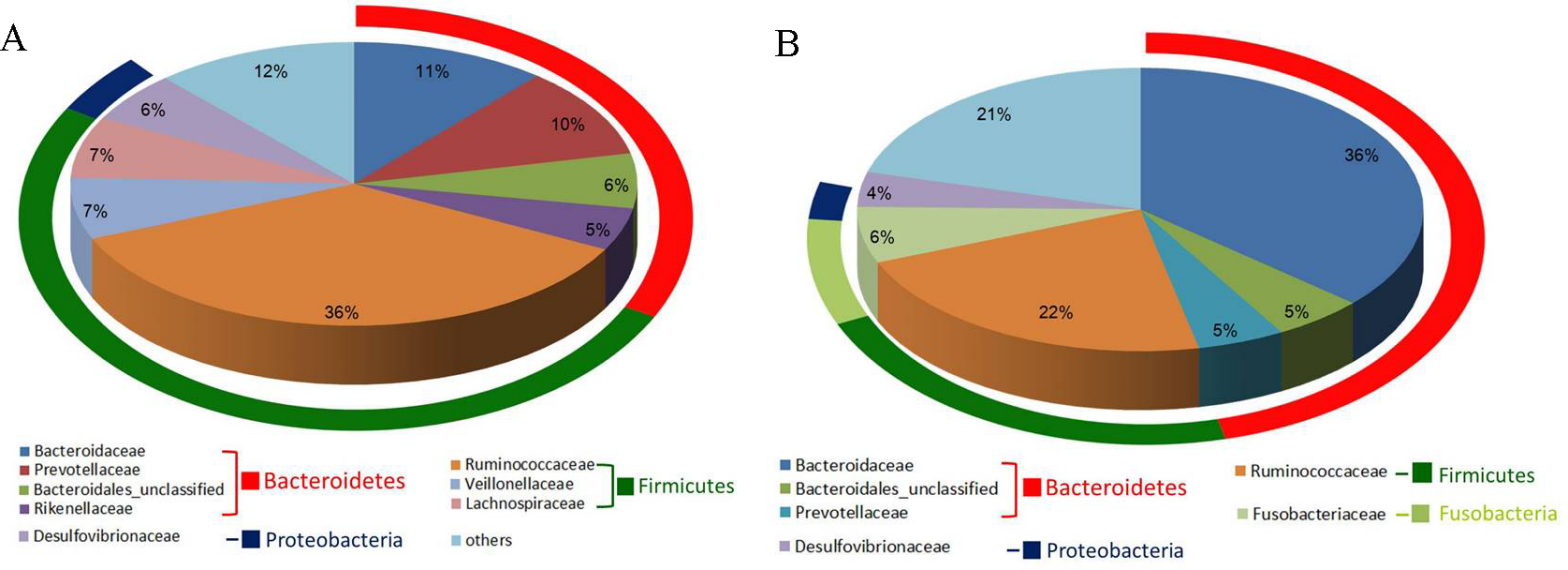
Pie charts of median percent values of bacterial families present in caecal samples of grass-fed and high-grain-fed geese (>3%). Rings represent the phylum (Bacteroidetes in green and Firmicutes in red) corresponding to each of the most frequently represented genera. A, caecal samples from grass-fed geese; B, caecal samples from high-grain-fed geese.

On comparing the caecal microbiota in geese receiving different diets, more than 80% of the sequences in all-grass-fed geese and high-grain-fed geese were found to belong to the two most populated bacterial phyla, namely Bacteroidetes and Firmicutes (**Fig 3**). However, the phylum Firmicutes exhibited higher abundance in the caecal samples of all-grass-fed geese (56.44% versus 31.18%, *P*<0.05), whereas Bacteroidetes were more abundant in high-grain-fed geese than in all-grass-fed geese (51.14% versus 34.69%, *P*>0.05). In addition, Fusobacteria (6.75%) and Deferribacteres (2.15%) was present in the high-grain-fed geese, while those were almost absent in all-grass-fed geese(**Table 2**). Interestingly, at the family level, the most highly abundance of the microbiota was Ruminococcaceae (Firmicutes) and Bacteroidaceae (Bacteroidetes) in all-grass-fed geese and high-grain-fed geese, respectively **(Fig 4)**.

**Table 2:** The different phyla of bacterial communities in caecal samples from all-grass-fed geese and high-grain-fed geese.

the phyla of bacterial communities	all-grass-fed geese(%)	high-grain-fed geese(%)	*P* value
Actinobacteria	0.240±0.179	0.302±0.139	0.688
Bacteroidetes	34.687±24.354	51.138±7.626	0.011
Candidate_division_TM7	0.000±0.000	0.002±0.003	0.024
Chloroflexi	0.001±0.002	0.002±0.003	0.348
Cyanobacteria	0.195±0.202	0.034±0.037	0.088
Deferribacteres	0.212±0.280	2.154±2.732	0.048
Elusimicrobia	0.080±0.074	0.031±0.032	0.268
Firmicutes	56.438±22.069	31.177±2.676	0.018
Fusobacteria	0.000±0.000	6.752±10.854	0.028
Lentisphaerae	0.421±0.747	0.742±0.868	0.424
Proteobacteria	6.399±4.388	4.848±2.746	0.198
SHA-109	0.000±0.000	0.330±0.660	0.024
Spirochaetae	0.535±0.876	0.288±0.214	0.074
Synergistetes	0.166±0.132	0.245±0.489	0.077
Tenericutes	0.357±0.274	0.166±0.139	0.275
Verrucomicrobia	0.268±0.249	1.788±2.339	0.039
WCHB1-60	0.000±0.000	0.002±0.003	0.024

Although a little difference was seen at the phylum level, 75 OTUs were very significantly different in abundance between geese on different diets (*P*<0.01) when comparing individual OTUs by ANOVA. Of these, 54 were Firmicutes, 14 Bacteroidetes, 3 Proteobacteria, 3 Actinobacteria and 1 Tenericutes. The abundance of the family Lachnospiraceae from the phyla Firmicutes was significantly higher in all-grass-fed geese than high-grain-fed geese. The relative abundance of 195 OTUs was significant difference between all grass-fed geese and high grain-fed geese (**S3 Table**).

In addition, a small number of OTUs were identified as making up the dominant community (the abundance accounting for over 1% of the total sequences at family level). It was clear that the 33 OTUs were shared in geese fed with different diets, which belonged mainly Bacteroidaceae and Ruminococcaceae families (**S4 Table**); and 3 OTUs and 8 OTUs were specific in grass-fed geese and high grain-fed geese, repectively (**S4 Table**).

**Fig 5:**
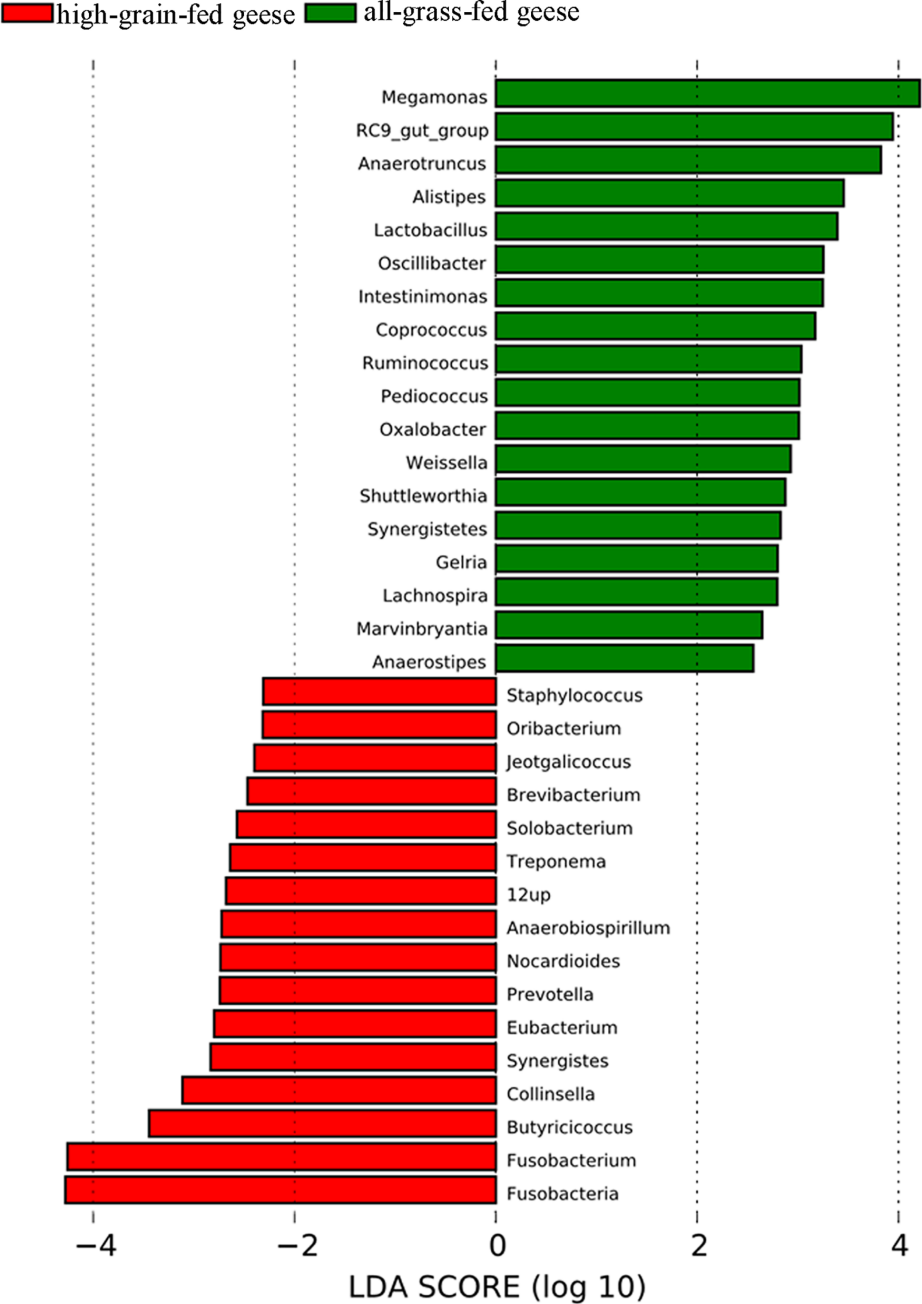
Chart representing LEfSe statistics in all-grass-fed and high-grain-fed geese. Bacterial taxa that were significantly different in abundance between all-grass-fed and high-grain-fed geese were identified by LEfSe using default parameters.

To further identify the differences in bacterial taxa between all-grass-fed and high-grain-fed geese, we performed LEfSe as well as non-parametric factorial Kruskal-Wall Prevote sum-rank test to detect differences in abundance 18 and 16 taxa were overrepresented in grass-fed geese (e.g. *Megamonas*, *Anaerotruncus*, *Alistipes*, *P*<0.05) and high-grain-fed geese (e.g. *Fusobacteria*, *Fusobacterium*, *Butyricicoccus*, *P*<0.05), respectively (**Fig 5**).

**Fig 6:**
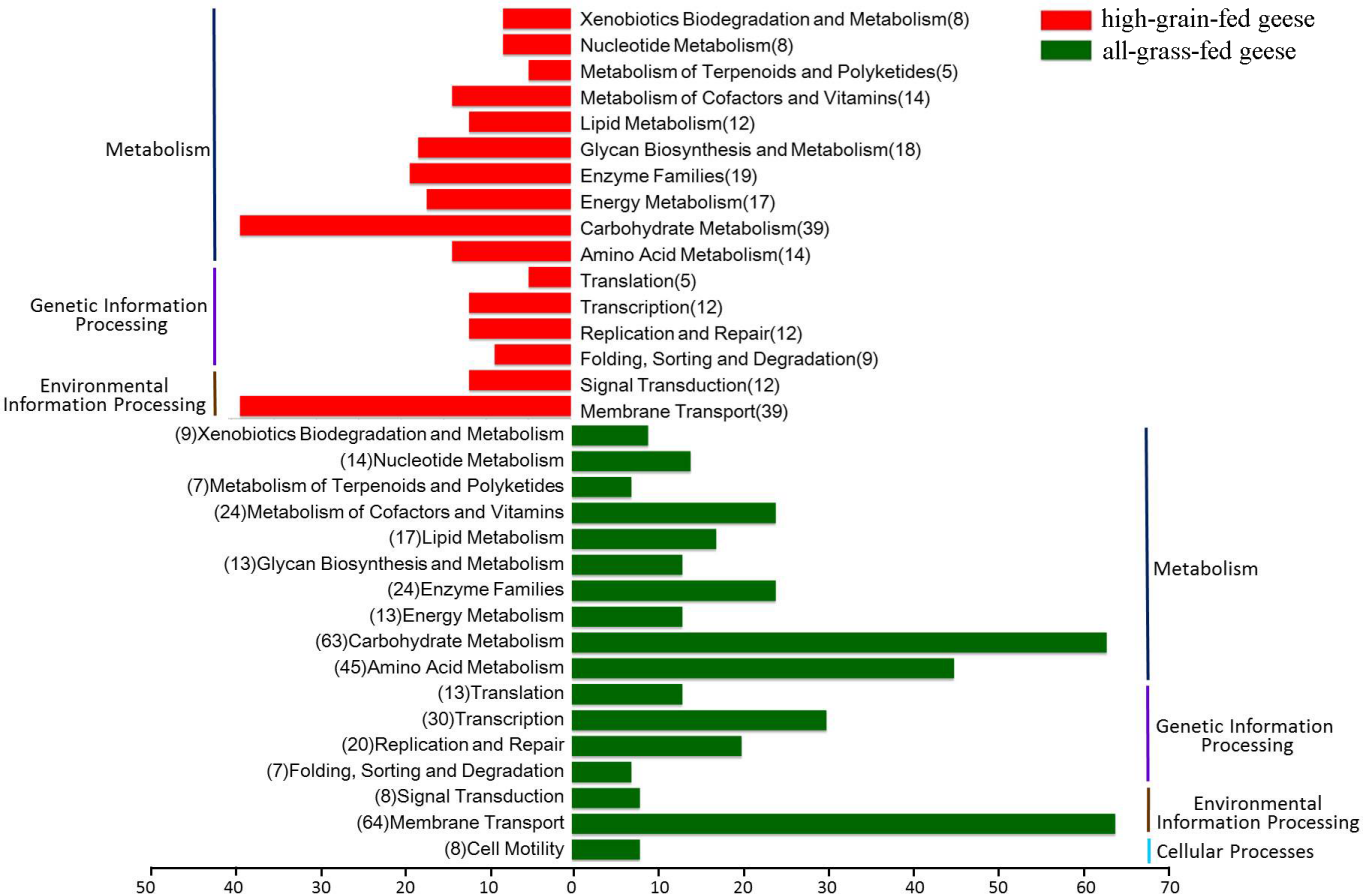
Enrichment analysis of KEGG to predict functions of gut microbiota between all-grass-fed geese and high-grain-fed geese. The total OTU distribution between groups was tested using bootstrap Mann-Whitney U-test with a cutoff of *P*<0.05. The brackets indicate the number of KEGG-related pathways.

We next used the PICRUSt tool to explore the functional profiles of the goose gut microbiota. The total OTUs were normalized by 16S rRNA gene copy number (http://picrust.github.io/picrust/tutorials/otu_picking.html) and their metagenomic functions predicted from the KEGG pathways. A total of 175 pathways were more abundant in all-grass-fed geese, while 117 pathways were significantly more abundant in high-grain-fed geese (*P*<0.01; **S5 Table**). Of these pathways, the greatest difference was observed among metabolic pathways between the two groups (**Fig 6**). For example, the KEGG Orthologies (KO) of ‘carbohydrate metabolism’, ‘metabolism of cofactors and vitamins’o and ‘amino acid metabolism’ were more represented in all-grass-fed geese than in high-grain-fed geese (63 versus 39).

## Discussion

The goose relies on the consumption of a certain amount of fibre that can be digested by the microbiota. Accumulating evidence emphasizes the tight interactions between the gut microbiota and the diet [30–33], but until now, no reports had explored the adaptive responses of the goose gut microbiota to grass feeding. In this study, we compared the characterization of bacterial communities between all-grass-fed geese and high-grain-fed geese. The goose microbiota was richer in OTUs and higher in alpha diversity when fed grass diet compared to grain diet. Previous studies have established that Shannon’s diversity index varied from 3 to 6 in the chicken caecal microbiota [34, 35], while varied from 7 to 9 in the caecal microbiota of rabbit [19], goat [33] and swine [36]. In this study, we found the average Shannon’s diversity index was about 4.2. Our comparative analysis found that the goose gut microbial diversity was similar to that of the chicken, but relatively less than mammalian species. We also noticed that the microbial diversity in all-grass-fed geese was significantly higher than that of high-grain-fed geese. In theory, a high level of diversity provides “functional redundancy” that helps maintain the stability of intestinal microbiota after environmental stress [37]. From this point, the gut microbiota may be more stable in all-grass-fed geese than high-grain-fed geese. We also detected large variations within the faecal microbiota from the Shannon and Simpson indices across different individuals; these variations may be distinct from those between grass-fed and grain-fed samples by unweighted UniFrac analysis. Similar results have been observed in chickens [38]. Hence, the results further confirmed that faecal microbiota do not provide a complete indication of the caecal community structure. In human studies, it has been suggested that faecal samples do not accurately reflected the population of the gut microbiota [39, 40]. In addition, Zhao et al have proved that the porcine microbial profile in feces was only the similar to those in the small intestine [41].

The goose is herbivorous; therefore, cellulose-degrading bacteria are particularly important for food degradation, especially when fed a high-cellulose diet. In the present work, regardless of diet, the dominant phyla appear to be Bacteroidetes and Firmicutes, which are typically enriched in other herbivores [42, 43]. However, the ratio of Firmicutes to Bacteroidetes differs in grass-fed geese and grain-fed geese, and a higher proportion of Firmicutes and a lower proportion of Bacteroidetes emerged in the grass-fed geese. In humans, the proportion of Firmicutes to Bacteroidetes (the F/B ratio) decreased on a low-calorie diet [44]. In our study, the grass diet was a low-calorie diet compared to the high-grain diet, but the F/B ratio of goose showed the opposite trend from that of humans. This may be due to differences in co-evolution between the gut microbiota of the host (human or avian) and diet. Surprisingly, Fusobacteria and Deferribacteres were highly abundance in high-grain-fed geese, while few in grass-fed geese, which was further confirmed by LEfSe analyses. These results suggest that Fusobacteria and Deferribacteres microbiota have adapted to digest the high-grain diet under the intensive systems. At the family level, Ruminococcaceae (Firmicutes) and Bacteroides (Bacteroidetes) were present exclusively in all-grass-fed geese and high-grain-fed geese, respectively. As a family of cellulose-degrading bacteria, the Ruminococcaceae has been widely confirmed to digest cellulose in human and the other animals [45–47].

Furthermore, of the 75 OTUs that were found to show significant differences in abundance between the birds of two dietary treatment geese, the majority are classified as Firmicutes (54 OTUs) with some Bacteroidetes (14 OTUs). Different OTUs belonging to the family Lachnospiraceae were found to be significantly higher in grass-fed geese. Lachnospiraceae are known butyrate producers [48], and perhaps the increased butyrate production helps to stimulate gastric cells proliferation and increase nutrients absorption [49, 50].

In the present study, the dominant bacterial community associated with the caecum in grain-fed geese(64.7%) was higher compared to grass-fed geese(47.3%), specifically in *Bacteroidaceae* which was more abundant in grain-fed geese (31.6%) than grass-fed geese (6.6%). The other microbiota community of goose from NCBI (ERR011376) also showed *Bacteroidaceae* was predominant bacterial community with an abundance of 43.03%. From the research above, Bacteroidetes may be considered as a dominant microbiota in goose. Zeng *et al*. (2015) studied the bacterial communities associated with body weight of Rex rabbits and found that the members of *Bacteroidaceae* were significantly enriched in high-weight rabbits [19]. In our study, we also measured the body weights of geese receiving different diets. The body weight of 70-day-old geese on the high-grain diet (2712.5±327.2g) was higher than that of geese on the grass diet (2630.0±309.3g)(**S6 Table**). Therefore, the members of *Bacteroidaceae* might contribute to the growth of animals.

With respect to metabolic pathways, a remarkable enrichment was observed in pathways relating to carbohydrate metabolism, metabolism of cofactors and vitamins, and amino acid metabolism in all-grass-fed geese. Due to the cellulose belonging to carbohydrate, those bacterial metabolic pathways might be more effective in cellulose digestion in the grass. PICRUSt provides important insight into bacterial community functions in the goose gut. Other omics approaches (e.g. transcriptomics and metabolomics) are desired to confirm these discoveries and improve our understanding of bacterial function in the goose gut.

### Conclusions

In summary, we presented comparative characterization of bacterial communities in geese fed all-grass or high-grain diets, and found The diets had significant effects in shaping the microbial community. Bacterial profile was primarily predominated by Firmicutes in all-grass-fed geese and Bacteroidetes in high-grain-fed geese, respectively. In addition, some specific microorgnisms including the Ruminococcaceae, Lachnospiraceae and Bacteroidaceae were identified which may associated with cellulose-degrading. Furthermore, the presence of the dominant communities composed predominantly of the Lachnospiraceae and Rikenellaceae, which were involved mainly in the carbohydrate metabolism in all-grass-fed geese. Thus, the study may provide foundation for further investigated the role of specific microbes in efficiency digestion products research of goose.

## Supporting information

S1 Table Sequences summary. (XLS)

S2 Table OTU and taxonomy information of caecum and faeces from grass-feed geese and high grain-feed geese. (XLS)

S3 Table Relative abundance of each OTU significant (P<0.05) for different diet and its classification. (XLS)

S4 Table Classification of the dominant bacterial community in the caecum from grass-fed geese and high grain-fed geese. (XLS)

S5 Table Prediction of KEGG pathways based on differentially represented OTUs. (XLS)

S6 Table The body weight of 70-day-old geese fed with all-grass diet and high-grain diet. (DOC)
