# Supplementary material for: Comparative characterization of bacterial communities in geese fed all-grass or high-grain diets
Source: PLoS One. 2017 Oct 3;12(10):e0185590. doi: 10.1371/journal.pone.0185590 (PMC5626430; doi:10.1371/journal.pone.0185590)
Supplement: S6 Table — (DOC) [file pone.0185590.s006.doc]

**S6 Table The body weight of 70-day-old geese fed with all-grass diet and high-grain diet**

| Diet | The body weight of 70-day-old geese(g) |
| --- | --- |
| All-grass diet | 2630.0±309.3^b^ |
| High-grain diet | 2712.5±327.2^a^ |

Note: Different lowercase letters indicate signiﬁcant differences (*P*<0.05).
